# Monitoring of erlotinib in pancreatic cancer patients during long-time administration and comparison to a physiologically based pharmacokinetic model

**DOI:** 10.1007/s00280-018-3545-4

**Published:** 2018-02-16

**Authors:** Andrea Gruber, Martin Czejka, Philipp Buchner, Marie Kitzmueller, Nairi Kirchbaumer Baroian, Christian Dittrich, Azra Sahmanovic Hrgovcic

**Affiliations:** 1 Division of Clinical Pharmacy and Diagnostics, Faculty of Life Sciences, University of Vienna, Althanstrasse 14, 1090 Vienna, Austria; 2 Applied Cancer Research - Institution for Translational Research Vienna (ACR-ITR VIEnna) and Ludwig Boltzmann Institute for Applied Cancer Research (LBI-ACR VIEnna), Centre for Oncology and Hematology, Kaiser Franz Josef-Spital, Bernardgasse 24/2, 1070 Vienna, Austria; 3 Austrian Society of Applied Pharmacokinetics, Krottenbachstrasse 184, 1190 Vienna, Austria

**Keywords:** Erlotinib, Long-time administration, Therapeutic drug monitoring, Interaction assessment, PBPK model

## Abstract

**Purpose:**

In this study, a therapeutic drug monitoring (TDM) of erlotinib in pancreatic cancer patients was performed over 50 weeks to reveal possible alterations in erlotinib plasma concentrations. Additionally, a physiologically based pharmacokinetic (PBPK) model was created to assess such variations in silico.

**Methods:**

Patients with advanced pancreatic cancer received a chemotherapeutic combination of 100 mg erlotinib q.d., 500–900 mg capecitabine b.d. and 5 mg/kg bevacizumab q.2wks. Samples were analyzed by HPLC and the results were compared to a PBPK model, built with the software GastroPlus™ and based on calculated and literature data.

**Results:**

The erlotinib plasma concentrations did not show any accumulation, but displayed a high inter-patient variability over the whole investigated period. Trough plasma concentrations ranged from 0.04 to 1.22 µg/ml after day 1 and from 0.01 to 2.4 µg/ml in the long-term assessment. 7% of the patients showed concentrations below the necessary activity threshold of 0.5 µg/ml during the first week. The impact of some co-variates on the pharmacokinetic parameters *C*_max_ and AUC_0–24_ were shown in a PBPK model, including food effects, changes in body weight, protein binding or liver function and the concomitant intake of gastric acid reducing agents (ARAs).

**Conclusion:**

This study presents the approach of combining TDM and PBPK modeling for erlotinib, a drug with a high interaction potential. TDM is an important method to monitor drugs with increased inter-patient variability, additionally, the PBPK model contributed valuable insights to the interaction mechanisms involved, resulting in an effective combination from a PK perspective to ensure a safe treatment.

## Introduction

Erlotinib (Tarceva^®^, OSI Pharmaceuticals, Melville, NY, USA; Roche, Basel, Switzerland; Genetech, South San Francisco, USA) is a potent and reversible inhibitor of the epidermal growth factor receptor (EGFR) tyrosine kinase and has been approved for the treatment of patients with metastatic non-small cell lung cancer and the treatment of patients with locally advanced, unresectable or metastatic pancreatic cancer, in combination with gemcitabine [1, 2]. It is available as a 25, 100 or 150 mg tablet and is given once daily at a fixed dose. The combination of erlotinib with capecitabine, bevacicumab or oxaliplatinum has been under investigation in the treatment of advanced pancreatic cancer [3, 4].

As a weak base, erlotinib quickly dissolves in the gastric acid of the stomach, but shows limited solubility at a pH above its pKa value of 5.4. Therefore, a physicochemical interaction with co-administered acid reducing agents (ARAs), increasing the gastric pH, is very likely to occur and has been reported before [5–7]. However, as a recent study showed, the negative influence of ARAs on erlotinib absorption can be diminished by drinking acidic beverages [8]. Erlotinib is well absorbed with mean peak plasma levels of 2–4 h after oral ingestion, resulting in an estimated bioavailability of 60% [1, 9]. Since the absorption of erlotinib can be influenced by food, its bioavailability is considered unpredictable after absorption to a fed state and can vary from 60 to 100% [1]. Hence, the intake of erlotinib is recommended to a minimum of 2 h after and 1 h before a meal. Due to its high lipophilicity, erlotinib is highly bound to plasma proteins at approximately 95%, mainly to albumin and α-1 acid glycoprotein [10], therefore the concomitant administration of drugs with high plasma protein binding can lead to altered unbound erlotinib plasma concentrations [11]. Erlotinib is primarily metabolized by CYP3A4 and to a minor extent by CYP1A2 and CYP1A1. A pre- or co-treatment with CYP3A4 inducers or inhibitors can alter the bioavailability of erlotinib and should thus be avoided during the treatment with erlotinib [12, 13]. Smokers should be advised to stop smoking during erlotinib therapy, due to a CYP induction and hence reduced plasma concentrations in comparison to non-smokers [14]. Gender aspects have been investigated, but resulted in a non-significant difference [15].

Due to the various possible pharmacokinetic interactions, plasma concentrations of erlotinib have been reported to show a high inter-patient variability [16]. In this study, the primary endpoint was to conduct a TDM of erlotinib over a long-time period to evaluate possible undesired changes in plasma concentrations. TDM is an effective tool in routine cancer care to reveal therapeutic interferences and to ensure that plasma concentrations of a drug are above the necessary threshold [17–19]. However, TDM is expensive and in case the influential co-variates on the plasma disposition are known, it would be easier and more economical to predict the concentration profile in a defined patient by a suitable software. In silico methods have shown to be of assistance in the drug development process since many years, but their support in later stages has been promoted only more recently [20–22]. Hence, the secondary objective of this study was to create a physiologically based pharmacokinetic (PBPK) model to predict a concentration–time curve with the software GastroPlus™ and use it to identify characteristics that may lead to altered erlotinib plasma concentrations, such as variations in body weight, liver function, certain co-medication or drug administration to a fasted or fed state.

## Methods

### Erlotinib study

#### Study population

Patients eligible for this phase 1b study suffered from histologically or cytologically documented adenocarcinoma of the pancreas with locally advanced not radically resectable or metastatic disease. Inclusion criteria for this study were ECOG performance status 0–2, age ≥ 18 years, life expectancy of ≥ 12 weeks, adequate bone marrow function (absolute neutrophil count (ANC) ≥ 1.5 × 10^9^/l, platelet count ≥ 100 × 10^9^/l, hemoglobin (Hb) ≥ 9 g/dl) adequate liver function (serum (total) bilirubin ≤ 3 × upper limit of normal (ULN), aspartate aminotransferase (AST), alanine aminotransferase (ALT) ≤ 2.5/5 × ULN (patients without/with liver metastases), albumin ≥ 25 g/l) and adequate renal function (serum creatinine ≤ 2 × ULN or creatinine clearance ≥ 50 ml/min). Patients must not have been treated for metastatic or locally advanced diseases, but were allowed prior adjuvant radiotherapy and previous adjuvant chemotherapy, excluding the three therapeutic agents used in this trial, capecitabine, erlotinib and bevacizumab. Amongst several further exclusion criteria, the most important were history or evidence of not controlled brain metastases or seizures, major surgical procedure planned within 28 days prior to study treatment, pregnant or lactating females or evidence of any disease or metabolic dysfunction that contradicts the use of the investigational drugs or puts the patient at high risk from treatment complications. All concomitant medication was reported and the intake of drugs inhibiting or inducing CYP3A4 was prohibited during the study, along with medication specifically contraindicated to one of the three study drugs [1, 23, 24]. All patients were asked to keep a diary during their treatment, containing co-medication and health status to retrace possible interactions and treatment failures.

#### Study design

The study was originally designed to evaluate the PK performance of erlotinib in the combination therapy of erlotinib, capecitabine and bevacizumab over a week, before the amendment for an evaluation of erlotinib plasma concentration over a longer period was approved by the Ethical Committee of the City of Vienna (vote EK 08-159-0908, EudraCT number 2008-004444-36) as a separate amendment to the clinical study protocol. Patients had been informed about the aim of this investigation and had given their written consent. The patients were divided into 4 dose levels, with constant erlotinib (100 mg, p.o., q.d.) and bevacizumab doses (5 mg/kg, i.v., q2wks), but different capecitabine doses (500, 650, 800, 900 mg, p.o., b.d.). Serial blood samples were obtained on day 1 pre-dose, 1, 2, 3, 4, 5, 6, 8 and 24 h after erlotinib ingestion. Blood samples on days 2–8 were drawn pre-dose and 4 h after administration of erlotinib. Further blood samples were obtained in the long-term evaluation once a week before erlotinib ingestion, hence 24 h after the last erlotinib dose. The *C*_trough_ value was selected as sampling time for the pharmacokinetic monitoring of erlotinib as recommended in literature [19].

#### Sample preparation, analysis and PK calculations

After removing the blood cells from the samples by centrifugation (10 min for 4000 rpm), erlotinib was separated from the plasma by solid phase extraction using Oasis^®^ HLB C18 cartridges. Erlotinib was quantified by a sensitive and selective, validated, reversed phase HPLC assay as described in the literature [25].

For the pharmacokinetic analysis of plasma concentration data on day 1, Phoenix WinNonlin version 6.2.1 software (Pharsight Corporation, a Certara™ company) was used to calculate the PK parameters *C*_peak_, *C*_trough_, *T*_max_, AUC_0–24_ as well as the volume of distribution (*V*_d_), total body clearance (Cl_tot_) and terminal half-life (*T*_1/2el_). For this purpose, the noncompartmental model 303 of the WinNonlin library was chosen. From day 2 until day 8, the trough and peak concentrations were analyzed, but only the trough concentrations were evaluated until the end of the study. The parameters were calculated as arithmetic mean ± SD and the range (min–max) was calculated for comparison to the simulation output.

The statistical evaluation of the plasma data was performed using the scientific software GraphPad Prism version 6.00 for Windows (GraphPad Software, La Jolla California USA).

### PBPK modeling

The erlotinib plasma concentration–time profile was created in a PBPK model using the GastroPlus™ software version 9.5. (Simulations Plus Inc., Lancaster, California, USA). A general description of the software is available in the user manual [26]. PBPK simulations differ from compartmental PK simulations in the calculation of diffusion coefficients for all compartments, for a more precise distribution of the compound into different tissues over time [27]. The physicochemical and absorption–distribution–metabolism–elimination (ADME) properties used in this model were calculated by the ADMET Predictor™ module of the software and are summarized in Table 1. Some parameters were optimized, using parameter sensitivity analysis (PSA) to obtain a good fit for the model.

**Table 1:** Input parameters for the erlotinib PBPK model in GastroPlus™

Parameter	Predicted value	Optimized value
Molecular weight (g/mol)	393.45	393.45
LogP (neutral)	3.13	2.7
Basic pKa	4.46	5.4
Intrinsic solubility (mg/ml)	0.078	0.0089
Solubility at pH = 2 (mg/ml)	22.44	0.40
Solubility factor	334.44	50.0
Permeability (cm/s × 10^−4^)	2.7	2.7
Fraction unbound in plasma (%)	4.57	4.57
Blood plasma ratio	0.71	0.71
Liver clearance (l/h)	40.0	4.0
FaSSIF (mg/ml)	0.003	0.003
FeSSIF (mg/ml)	0.117	0.7
Solubilization ratio	1.03E+04	8.01E+04
Particle radius/diameter (µm)	25/50	15/30
Mean precipitation time (s)	900	100

Solubility factor: ratio of the solubility of ionized to unionized drug; FaSSIF: compound solubility in intestinal fluid in fasted state; FeSSIF: compound solubility in intestinal fluid in fed state; solubilization ratio: effect of bile salt concentration in FaSSIF and FeSSIF media on solubility of the compound

#### Input data for the model

Tarceva^®^ 100 mg tablets contain 109.3 mg erlotinib hydrochloride, which is equal to 100 mg erlotinib free base [1]. The properties of the erlotinib base were used as input parameters for the PBPK model. Erlotinib is a weak base and a lipophilic compound, with a good intestinal permeability. The free base is only very slightly soluble in water, but the solubility of the hydrochloride salt is higher at a lower pH, indicating that the drug will easily dissolve in the acidic environment of the stomach. With the overall low solubility and a high permeability, the drug is considered a Biopharmaceutics Classification System (BCS) class II compound [28].

#### Modeling strategy

The general workflow of PBPK modeling has been described in many publications and tutorials [29–31]. The preliminary model in this case was based solely on the physicochemical data from the ADMET Predictor™ module of GastroPlus™, using a human PBPK model of a standard 30-year old, healthy man, which was subsequently changed to a man of 60 years and 60 kg, corresponding to the study population. The physicochemical parameters logP, pKa and intrinsic solubility were updated based on literature data [1, 32]. The best suited distribution model was the Lukacova model, which was chosen according to the properties of the compound for the perfusion-limited tissue distribution coefficients of erlotinib [26]. The liver clearance, calculated by ADMET Predictor™ was implemented and adjusted to match the observed Cl_tot_ in the population. Since the majority of erlotinib is metabolized in the liver, the gut metabolism was excluded in this model. Concerning the oral absorption modeling, the dissolution was best described by the Johnson model and the particle size was adapted to depict the quick dissolution of erlotinib in the acidic environment of the stomach. The selected gut physiology calculation method was the human-physiological-fasted model with the Opt logD Model SA/V 6.1 for the calculation of the absorption scale factors. Ultimately, the solubilization ratio (SR) and the mean precipitation time were optimized by PSA to fit the oral absorption. The SR is calculated by GastroPlus™ according to the solubility of the compound in simulated intestinal fluids in fasted (FaSSIF) and fed state (FeSSIF) and gives an idea on how much additional solubility can be gained through the increased intestinal bile salt concentration in a fed state [26].

The PBPK model was calculated as a single and a population simulation, whereas the population simulation was set up with 25 American patients of 50–70 years and was used as comparison to the erlotinib plasma concentrations of the population studied. The simulation output was formulated as arithmetic mean with a 90% confidence interval (CI) and the corresponding range (min–max). The single simulation of a standard patient of 60 years and 60 kg, receiving erlotinib at a fasted state was used to evaluate the impact of possible co-variates on the plasma concentration of erlotinib. The selected co-variates include ingestion of erlotinib at a fasted or fed state, changes in body weight, protein binding and liver function and the influence of a concomitant use of ARAs during the erlotinib therapy. For the fed state, the absorption model human-physiological-fed was chosen and the liver blood flow was adapted [26]. Differences in body weight, liver clearance and protein binding were considered by modifying the according parameters. The concomitant intake of ARAs was modeled by changing the stomach pH from 1.3 to 5.0 and increasing the transit time from 0.25 to 0.5 h [33].

## Results

### Patients characteristics

26 Patients with advanced, metastatic pancreatic cancer were selected to participate in the first week of the study. A subsequent set of 10 out of the 26 patients was chosen according to the selection criteria specified in the Methods section, to participate in the amendment of the study for a longer period. The demographics of the patients are listed in Table 2. Since there was no sign of interference from the different capecitabine doses on erlotinib plasma concentrations, the four dose levels with varying capecitabine doses, but constant bevacizumab and erlotinib doses, were pooled for the pharmacokinetic calculations. All 26 patients completed the first week of the study, but only 2 out of 10 patients completed the therapy up to 50 weeks; 8 patients were discontinued due to progression of the disease. The patients were checked upon every week concerning their performance status and 3 patients were therefore temporarily discontinued during the 50 weeks, because of toxicity and side effects.

**Table 2:** Patient demographics

Characteristics	Week 1	Week 2–50
	(*n* = 26)	(*n* = 10)
Gender *n* (%)		
Female	14 (53.8%)	6 (60%)
Male	12 (46.2%)	4 (40%)
Age (years)		
Median (min–max)	65.5 (47–80)	65.5 (55–74)
Body weight (kg)		
Median (min–max)	68.5 (44–97)	63.0 (44–84)
Body height (cm)		
Median (min–max)	170.0 (156.0–186.0)	170.0 (156.0–186.0)
Body surface (m^2^)		
Median (min–max)	1.79 (1.36–2.22)	1.69 (1.36–2.06)

### Long time performance

The analyses of the plasma samples on day 1 showed a mean erlotinib peak concentration of 0.84 µg/ml at 2 h after administration, but revealed a high variability of *C*_peak,_ from 0.21 to 1.82 µg/ml. The mean trough concentration at 24 h after ingestion was 0.33 µg/ml in a range of 0.04–1.22 µg/ml. The AUC_0–24_ ranged from 1.23 to 36.37 µg h/ml with a mean value of 12.47 µg h/ml. With a bioavailability of 60% [1, 11], the calculated *V*_d_ is 97.86 l, the Cl_tot_ is 5.51 l/h and the mean *T*_1/2el_ is 18.72 h. The key PK parameters are summarized in Table 3.

**Table 3:** Observed and simulated PK parameters of erlotinib for day 1, 3 and 6

Time	Parameters	Dimension	Observed			Simulated		
			Mean^a^±SD	Min–max	*N*	Mean^a^	Min–max	*N*
Day 1	*C*_peak_	µg/ml	0.84 ± 0.54	0.21–1.82	26	0.78	0.60–0.99	25
	*C*_last_	µg/ml	0.33 ± 0.29	0.04–1.22	26	0.30	0.29–0.31	25
	*T*_max_	h	2.00	1.00–8.00	26	1.89	1.20–2.90	25
	AUC_0–24_	µg-h/ml	12.47 ± 9.38	1.23–36.37	26	11.47	9.57–14.07	25
	*T*_1/2el_	h	18.72 ± 13.10	4.10–59.00	26	10.90	nc	25
	*V*_d_	l	97.86 ± 61.72	18.00–268.09	26	67.80	nc	25
	Cl_tot_	l/h	5.51 ± 5.73	0.47–30.09	26	4.30	nc	25
Day 3	*C*_trough_	µg/ml	0.45 ± 0.37	0.003–1.51	26	0.44	0.40–0.48	25
	*C*_peak_	µg/ml	1.04 ± 0.62	0.19–2.99	26	1.04	0.98–1.10	25
Day 6	*C*_trough_	µg/ml	0.72 ± 0.53	0.05–2.20	26	0.51	0.46–0.56	25
	*C*_peak_	µg/ml	1.38 ± 0.66	0.11–3.24	26	1.09	1.02–1.15	25

*C*_*peak*_ peak plasma concentration, *C*_*last*_ plasma concentration of the last analyzed sample, *T*_*max*_ time of peak plasma concentration, *AUC*_*0–24*_ area under the curve for the time 0–24 h, *T*_*1*/*2el*_ terminal elimination half-life, *V*_*d*_ volume of distribution, *Cl*_*tot*_ total body clearance, *C*_*trough*_ plasma concentration before subsequent drug ingestion, *nc* not calculable

^a^*T*_max_ data are expressed as median, all other PK parameters are calculated as arithmetic mean

Based on the analyses of the first day, the parameters *C*_peak_ and *C*_trough_ were expected to illustrate a continuously high variation in the first week. The steady state was reached 6 days after the first administration with a mean *C*_peak_ of 1.38 µg/ml in a range of 0.11–3.24 µg/ml. The mean *C*_trough_ value after 6 days was 0.72 µg/ml and varied from 0.05 to 2.27 µg/ml. Although the mean *C*_peak_ and *C*_trough_ values were above the activity threshold of 0.5 µg/ml, 10 of the 26 patients did not reach the threshold within 24 h after the first administration and 2 of the 26 patients never reached the threshold in the first week of erlotinib therapy. The administration of capecitabine and bevacizumab was continued for treatment purpose.

In the long-term monitoring, the erlotinib plasma concentrations did not show any significant accumulation in the blood over the whole investigated period. However, the high variability persisted throughout the rest of the study (Table 4). Mean erlotinib trough concentrations in the long-term study were calculated for each patient and ranged from 0.06 to 1.78 µg/ml. The long-term analysis showed *C*_trough_ values below the activity threshold for 5 of the 10 patients, who all received ARAs concomitantly. An unpaired *t* test demonstrated a statistically significant difference (*P* < 0.002) between the ARA and non-ARA group in the calculated trough concentrations.

**Table 4:** Mean trough concentrations ± SD (µg/ml) during long time administration of 100 mg erlotinib q.d.

Patients	Weeks	*N*	*C*_trough_^a^	Min–max
Pat.1	1–15	10	1.67 ± 0.44	0.77–2.20
Pat.2^b^	1–9	9	0.16 ± 0.04	0.12–0.23
Pat.3	1–50	50	0.89 ± 0.68	0.09–2.68
Pat.4	1–8	7	0.66 ± 0.27	0.14–1.06
Pat.5^b^	1–4	4	0.37 ± 0.09	0.25–0.46
Pat.6	1–8	8	1.78 ± 0.74	0.73–3.06
Pat.7	1–9	9	1.20 ± 0.51	0.31–1.80
Pat.8^b^	1–16	16	0.27 ± 0.38	0.02–1.31
Pat.9^b^	1–50	47	0.06 ± 0.04	0.01–0.16
Pat.10^b^	1–11	11	0.16 ± 0.06	0.07–0.31

^a^All mean *C*_trough_ values are calculated as arithmetic mean

^b^Patients with co-medication of acid reducing agents

### PBPK basic model

**Fig. 1:**
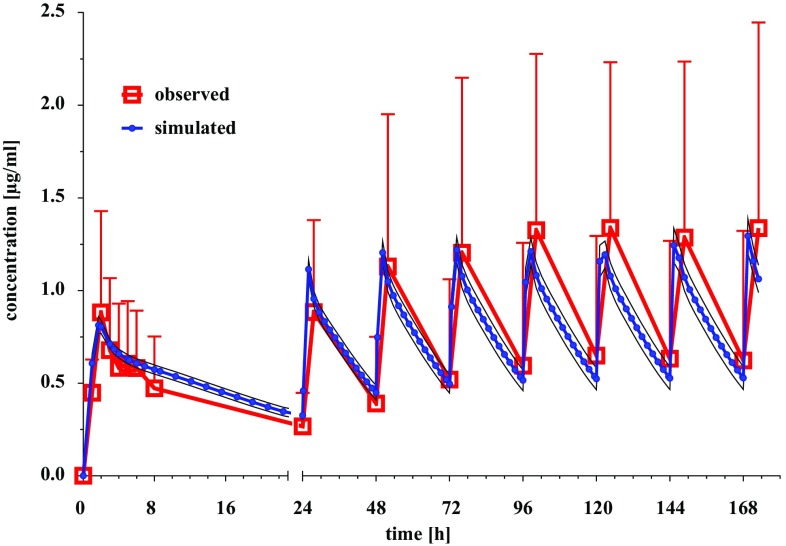
Comparison of the observed mean erlotinib plasma concentration (± SD) for the days 1–8, to the simulated mean erlotinib plasma concentration (± 90% CI), calculated by the population simulation model of GastroPlus™

The workflow for building, optimizing and verifying the erlotinib model is described in the “Methods” section. The preliminary model resulted in an underprediction of *C*_max_ and AUC_0–24_ by 37 and 90%, respectively, corresponding to the observed values; hence a further optimization was necessary. Therefore, the physicochemical parameters logP, pKa, intrinsic solubility and the maximum solubility of the hydrochloride salt as well as the physiology settings and the liver clearance were adjusted. The optimized model was evaluated with in vivo data of an i.v. application of 100 mg erlotinib and resulted in an underprediction of 20% in AUC_0–24_ and an overprediction of 12% for the *C*_peak_, compared to the mean parameter values of the study [34]. For the modeling of the oral absorption, the dissolution and absorption models were implemented and the particle size, SR and the mean precipitation time were modified. The population simulation resulted in a slight underprediction of AUC_0–24_ by 9% and *C*_max_ was underpredicted by 13% in comparison to the mean plasma concentration of the erlotinib study, but both values were still well within the observed concentration range as can be examined in Fig. 1.

### Co-variates

**Fig. 2:**
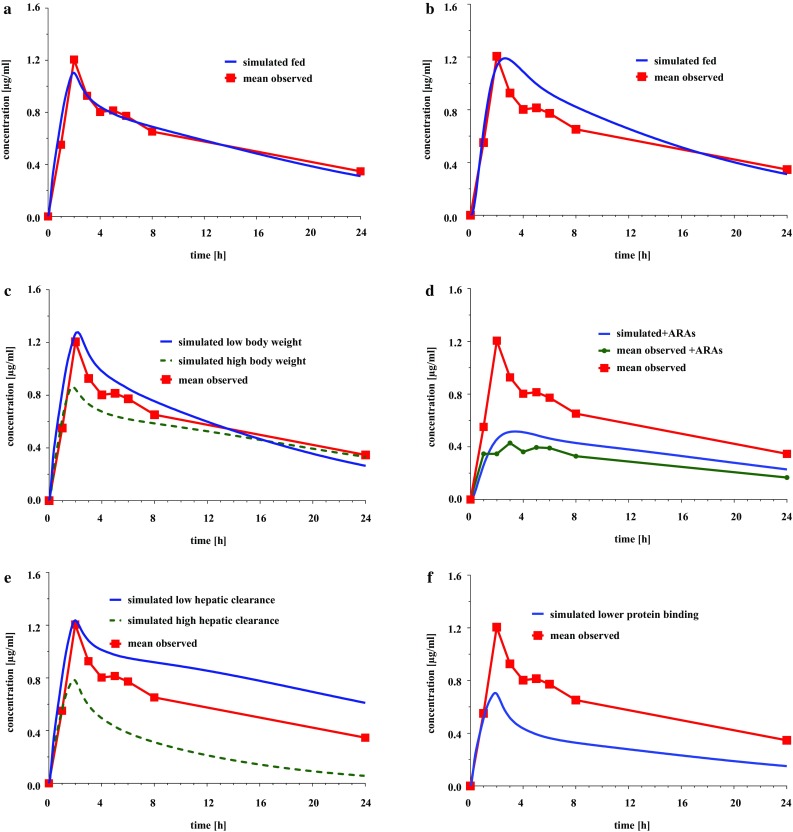
Simulated impact of co-variates on erlotinib plasma concentration vs observed mean concentration. **a** Standard patient without co-variates, **b** ingestion of erlotinib at a fed state, **c** patients with elevated (85 kg) and reduced (45 kg) body weight, **d** concentrations with co-medication of acid reducing agents (ARAs) vs observed concentration without ARAs, **e** patients with elevated (10 l/h) and reduced (2 l/h) hepatic clearance, **f** patients with decreased protein binding (10% fraction unbound)

Figure 2 illustrates the effect of possible co-variates on erlotinib plasma concentration. Insert a displays the comparison of the mean observed concentration in patients, who received erlotinib to a fasted state, without any relevant co-medication to the predicted GastroPlus™ single simulation, matching the settings of the standard patient of the study. *C*_peak_ differed by 8.5% but AUC_0–24_ achieved a 100% fit to the observed values, concluding that the prediction can be used for further modifications. The administration of erlotinib at a fed state, resulted in an increased bioavailability and AUC [1], as could be shown in insert b for the AUC_0–24_, which increased by 12% from 14.1 to 15.8 µg h/ml. Body weight has no impact on the total body clearance of the drug [35], but has a potential effect on the *C*_max_ and the *V*_d_, as shown in insert c. A reduced body weight resulted in a 16% higher *C*_max_ and a 25% lower *V*_d_ and the increased body weight caused a decrease in *C*_max_ by 20% and higher distribution into the tissue by 42%. Insert d depicts the difference in *C*_max_ and AUC_0–24_, due to co-administered ARAs, which reduced the AUC_0–24_ by 39% and *C*_max_ by 49% in the simulation, compared to a reduction by 52 and 56% respectively, in the study population. The physicochemical drug–drug interaction has been reported [7–9] and the PBPK model, as well as the analyses of the plasma samples of patients who received ARA co-medication in this study, support these findings. Changes in the hepatic clearance are displayed in insert e, from an elevated hepatic clearance of 10 l/h to a reduced liver clearance of 2 l/h. This range was obtained from the PK analyses of the plasma concentrations of the study. In the simulated high hepatic clearance patient, *C*_max_ decreased by 26% and AUC_0–24_ by 55%, and in the low hepatic clearance patient *C*_max_ increased by 12% and AUC_0–24_ by 41% in comparison to the values of the average patient. The fraction unbound of erlotinib in plasma was estimated to be 4.6%, but when changed to 10%, due to a possible pharmacokinetic interaction with a strongly protein-bound co-medication, and therefore a higher fraction unbound of erlotinib, as shown in insert f, the plasma concentration did not result as expected in a higher *C*_max_ and AUC_0–24_, but was distributed to a higher extent into adipose and liver tissue, and resulted in a higher Cl_tot_ of 7.66 l/h and lower *C*_max_ and AUC_0–24_ by 33 and 49%, respectively.

## Discussion

TDM has shown to be beneficial in oncological patients to ensure a safe and effective treatment, especially when drugs with high inter-patient variabilities are used. To date, TDM of erlotinib has been reported up to a maximum of 30 days [4, 36]. In this study, it is the first time that erlotinib levels have been monitored closely for 1 week and further once a week for up to 50 weeks. The daily administration of erlotinib over a long-time period did not lead to drug accumulation in the central compartment. Steady state was reached within 6 days after the first erlotinib ingestion, but the variability in plasma concentrations remained high throughout the study. However, based solely on the limited knowledge about the study population, the co-variates, influencing the plasma concentrations, were difficult to deduce.

PBPK models have gained more importance with the increased progress of their features and are useful in many stages of drug development. In this case, the PBPK model was built to demonstrate the influence of co-variates on the erlotinib plasma concentration. The administration of TKIs to a fed state has often been discussed to increase the AUC and bioavailability [12], which was shown accordingly in the model. The influence of body weight on the distribution of a lipophilic compound such as erlotinib was also shown and matched our expectations, as did the differences in hepatic clearance. When a higher or lower liver clearance rate was implemented, the elimination changed accordingly. However, the simulation of an increased unbound fraction of erlotinib did not result in the expected elevated plasma concentration, but in a higher distribution into adipose and liver tissue and therefore an increased elimination and lower plasma concentration. The biggest influence though seemed to come from the concomitant intake of ARAs, which has been reported before in patients [7, 8] and healthy subjects [9]. The decreased AUC_0–24_ and *C*_peak_ levels often result in an ineffective treatment below the activity threshold. Patients receiving both, erlotinib and ARA were advised to terminate the use of ARAs, but due to gastrointestinal side effects, some patients continued a combined intake.

In conclusion, a PBPK model can demonstrate effects of co-variates that are known in advance. However, although assumptions about possible interactions can be drawn from other drugs of the same class of compounds, the list might not be complete and some influences might still be unrevealed. Therefore, a TDM is nonetheless recommended for drugs with a high interaction profile and a narrow therapeutic window and cannot be replaced entirely by in silico predictions. From a PK point of view, PBPK modeling combined with TDM represents a new strategy to evaluate the therapy of drugs with high inter-patient variability.
